# P-370. Impact of a Multidisciplinary Intervention to Reduce Infection after Prostate Biopsy at a Veterans Affairs Medical Center

**DOI:** 10.1093/ofid/ofae631.571

**Published:** 2025-01-29

**Authors:** Melissa Whitman, Roy T Sabo, Angela Eckert, Matthew M Hitchcock, Emily Hill, Sarah Krzastek, baruch grob, John Markley

**Affiliations:** Virginia Commonwealth University, Richmond, Virginia; Virginia Commonwealth University, Richmond, Virginia; Central Virginia VA Health Care System, Amelia, Virginia; Richmond VA Medical Center, Richmond, Virginia; Central Virginia VA Health Care System, Amelia, Virginia; Richmond Veteran Affairs Medical Center, Richmond, Virginia; Richmond Veterans Affairs Medical Center, Richmond, Virginia; Hunter Holmes VA / Virginia Commonwealth University, Richmond, Virginia

## Abstract

**Background:**

Infection after prostate biopsy (IAPB) occurs in 2-6% of cases. Due to fluoroquinolone resistance at our facility, preprocedural prophylaxis for prostate biopsy was switched from Ciprofloxacin to Fosfomycin in 2017. However, the rates of IAPB at our facility increased from 0.93% to 4.27% between 2021 and 2022 (Figure 1). Herein we describe the impact of a series of interventions aimed at reducing the IAPB rate.

Table One

**Figure:**
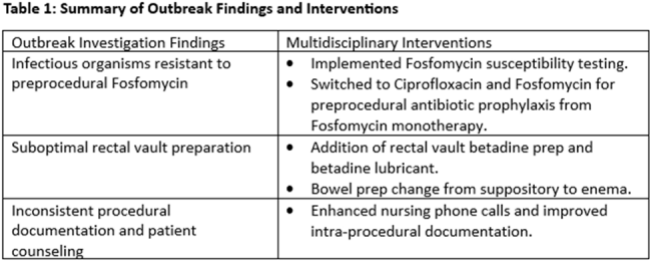

**Methods:**

A multidisciplinary workgroup including infection control, antimicrobial stewardship, nursing, and urology was convened in February 2022. An outbreak investigation ensued utilizing active surveillance, direct procedural observation, and chart review for epidemiologic commonalities and appropriateness antibiotics (Table 1). Based on these findings, several interventions were implemented (Table 1). IAPB rates were modelled over time using a generalized additive spline model with 2 knots. Data analysis took place between Oct 2018 to Sept 2023 using SAS Statistical Software (version 9.4. Cary, NC, USA).

Figure One

**Figure:**
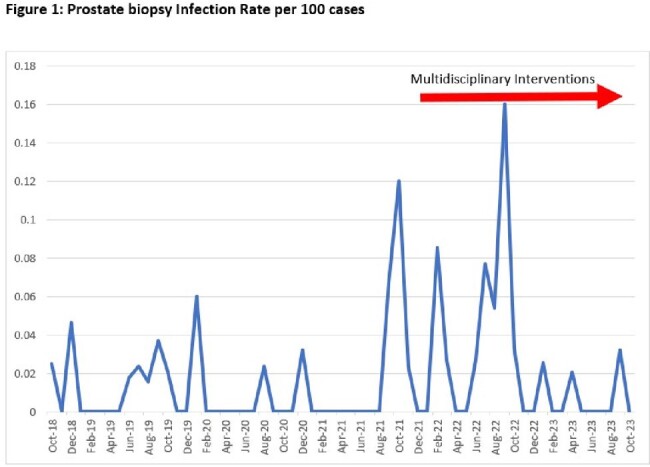

**Results:**

A total of 2,295 prostate biopsies and 37 infections were identified from Oct 2018 to Sept 2023. Numerous bacterial pathogens were implicated, and no point source of infection was identified. Multilocus sequence-typing of three Klebsiella pneumoniae isolates did not suggest a single-organism outbreak. Pseudomonas was implicated in 4 infections. Fosfomycin minimum inhibitory concentrations were elevated in 6/9 isolates available for testing (range 32 to >1024 ug/mL). Three periods with different infection rates were identified (Figure 2). Mean infection rates were 1% (0.6%-1.7%) for the first period (Oct 2018 to Aug 2021), 4.4% (2.8%-6.7%) for the second (Sept 2021 to Oct 2022), and 0.7% (0.2%-2.0%) for the third (Nov 2022 to Sept 2023). Periods one and three had statistically significant lower infection rates than period two (p=0.0002 and p=0.004, respectively).

**Figure:**
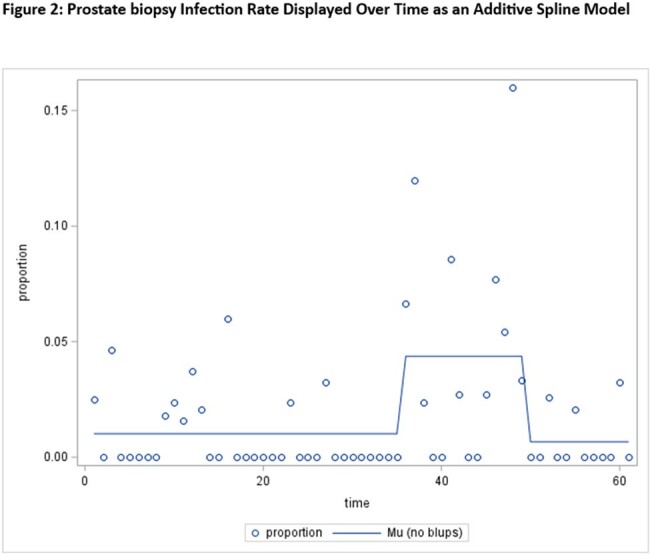

**Conclusion:**

A multidisciplinary workgroup applying a series of interventions was associated with a significant reduction in IABP. In the context of evolving antimicrobial resistance and variable procedural practices, elevated IABP should prompt a systematic evaluation of antimicrobial prophylaxis and procedural practices.

**Disclosures:**

**All Authors**: No reported disclosures

